# Sea Star Wasting Disease in *Asterias forbesi* along the Atlantic Coast of North America

**DOI:** 10.1371/journal.pone.0188523

**Published:** 2017-12-11

**Authors:** Caitlin Bucci, Madison Francoeur, Jillon McGreal, Roxanna Smolowitz, Vanesa Zazueta-Novoa, Gary M. Wessel, Marta Gomez-Chiarri

**Affiliations:** 1 Department of Fisheries, Animal and Veterinary Science, University of Rhode Island, Kingston, Rhode Island, United States of America; 2 Department of Biology and Marine Biology, Roger Williams University, Bristol, Rhode Island, United States of America; 3 Department of Molecular and Cellular Biology, Brown University, Providence, Rhode Island, United States of America; Linnaeus University, SWEDEN

## Abstract

As keystone species, sea stars serve to maintain biodiversity and species distribution through trophic level interactions in marine ecosystems. Recently, Sea Star Wasting Disease (SSWD) has caused widespread mass mortality in several sea star species from the Pacific Coast of the United States of America (USA) and *Asterias forbesi* on the Atlantic Coast. A densovirus, named Sea Star associated Densovirus (SSaDV), has been associated with the wasting disease in Pacific Coast sea stars, and limited samples of *A*. *forbesi*. The goal of this research is to examine the pathogenesis of SSWD in *A*. *forbesi* on the Atlantic Coast of the USA and to determine if SSaDV is associated with the wasting disease in this species. Histological examination of *A*. *forbesi* tissues affected with SSWD showed cuticle loss, vacuolation and necrosis of epidermal cells, and oedema of the dermis, but no consistent evidence indicating the cause of the lesions. Challenge experiments by cohabitation and immersion in infected water suggest that the cause of SSWD is viral in nature, as filtration (0.22 μm) of water from tanks with sea stars exhibiting SSWD did not prevent the transmission and progression of the disease. Death of challenged sea stars occurred 7–10 d after exposure to infected water or sea stars, and the infectivity crossed species (*A*. *forbesi* and *Pateria miniata*) with equal penetrance. Of the 48 stars tested by quantitative real time PCR, 29 (60%) were positive for the SSaDV VP1 gene. These stars represent field-collected sea stars from all geographical regions (South Carolina to Maine) in 2012–2015, as well as stars exposed to infected stars or water from affected tanks. However, a clear association between the presence of SSaDV and SSWD signs in experimental and field-collected *A*. *forbesi* was not found in this study.

## Introduction

Forbes sea star, *Asterias forbesi*, is an intertidal (<30m deep) asteroid found along the Atlantic Coast of the United States (USA) from Maine to the Gulf of Mexico. These stars are broadcast spawners, meaning external fertilization creates a free-swimming bipinnaria larval stage. Approximately three weeks after fertilization, the larvae metamorphose into the pentaradially symmetrical adults [1]. Sea stars, including *A*. *forbesi*, act as keystone species, maintaining ecosystem diversity and stable population densities of other marine invertebrates through predation and other trophic and food web interactions [2]. For example, in 1999 a sharp decrease in the abundance of *A*. *forbesi* in Raritan Bay was associated with a marked increase in the abundance of northern quahogs [3]. Due to their role as keystone predators, epizootics affecting echinoderm species can have significant effects on ecosystem stability. When mass-mortalities of the sea urchin *Diadema antillarum*, an important grazer of coral reefs, occurred in the Caribbean in the 1980’s, the community faced an extreme shift in population dynamics. Without the sea urchins to graze the reefs, microalgae began to overgrow the coral and dense algal mats eventually smothered the reefs [4].

Over the last four decades prior to 2012, several mass mortality events in sea star populations along the Pacific Coast of the USA have been reported [5,6]. Sea star wasting was first reported in the sunflower star, *Heliaster kubiniji* along the Gulf of California in June 1978. These stars exhibited white lesions on the aboral surface, followed by fragmentation and death [5]. In the summer of 1997, more than ten species, including *Asterina (*now *Pateria*) *miniata* and *Pisaster giganteus*, were similarly affected in the Channel Islands (California). These sea stars exhibited similar signs of wasting, including loss of turgor, white lesions on the aboral surface, and ultimately fragmentation and death [7].

In the spring of 2012, anecdotal reports from fishers, aquaculturists, and sea star collectors for aquariums and educational institutions indicated increased incidence of health issues in sea stars on the North Atlantic Coast of the USA (personal communications). By 2013, over 20 species of stars on both the Atlantic and Pacific Coasts of the United States were reportedly affected by what was termed “Sea Star Wasting Disease” (SSWD) [8]. Affected stars were flaccid or showed curled limbs and the aboral surface was indistinct with a mucoid appearance. Stars often dropped limbs, followed by disintegration of the central disc and body wall [8]. The rapid spread of SSWD, as well as the widespread geographical distribution and species affected, makes it of great concern to individuals and organizations interested in the stewardship of biodiversity and the conservation of ocean resources [9–11].

A densovirus, named Sea Star Associated Densovirus (SSaDV), was reportedly linked to SSWD in more than 20 species on the Pacific Coast of the US, in 9 of 14 samples of *A*. *forbesi* tested from the Atlantic Coast [8], and in 23 of 48 samples collected in February 2013 from a location in Rhode Island (RI) affected by SSWD [12]. Another virus, named *Asterias forbesi-*associated circular virus (AfaCV) was discovered in a few *A*. *forbesi* with signs of SSWD collected in 2011, but the lower prevalence of this virus (10% compared 48% for SSaDV) in the 2013 sample from a RI population suggested no association with SSWD [12]. Densoviruses are a genus of the *Parvoviridae* family, which includes single-stranded, non-enveloped, DNA viruses infecting invertebrates such as insects and crustaceans. Prevalence of SSaDV in Pacific Coast sea stars was found to be higher in moribund than in healthy appearing individuals, and stars with higher viral loads were more likely to show clinical signs of SSWD. Viral load also increased as disease signs progressed, suggesting a potential relationship between SSaDV and SSWD [8]. However, the association between viral presence and SSWD was not definitive, as some diseased animals contained no detectable densovirus. The lack of sea star cell lines, and the low possibility of generating pure virus, encumbers fulfilling Koch’s Postulates for SSWD [13].

Due to the small sample size of Atlantic Coast sea stars tested so far [8, 12], the association between SSWD and SSaDV on the Atlantic Coast of the USA is still unclear. Moreover, the epizootiology and pathogenesis of SSWD in *Asterias* spp. has not been well characterized. The overall goal of this research is to assess the pathogenesis of Sea Star Wasting Disease (SSWD) in *A*. *forbesi* along the Atlantic Coast of the USA. In this study, we: 1) describe the clinical signs of SSWD in *A*. *forbesi* and a few other echinoderms from aquariums and field populations along the Atlantic Coast of the USA between 2012 and 2015; 2) investigate the pathogenesis of the disease and potential mode(s) of transmission using challenge experiments; and 3) assess the presence and abundance of SSaDV in *A*. *forbesi* samples from experimental challenges and field populations.

## Materials and methods

### Specimen collection and animal husbandry

Sample collection in Rhode Island was performed under a scientific collection permit by the Rhode Island Department of Environmental Management. Samples of sea stars from other locations in USA and Canada were collected under the institutional permits for the collecting institutions (Table 1 and S1 Table). This research did not involve the use of vertebrate or endangered species. Forbes sea stars (*A*. *forbesi*) were collected along the coast of Rhode Island between August 2013 and April 2015 from intertidal and subtidal habitats in Narragansett Bay, including the pier at the University of Rhode Island Graduate School of Oceanography (GSO; GPS coordinates 41°26’56.0” N, 71°24’00.4”W; n = 37) and Beavertail State Park (41°29’32.7”, 71°25’11.1”W; n = 17). Stars were classified as lacking or showing clinical signs of Sea Star Wasting Disease (SSWD) based on the following gross signs: a) loss of turgor pressure, b) mucoid appearance of the aboral surface, c) white, variably sized necrotizing lesions along rays of the body, d) macroscopically distinct borders, and e) limb autotomy. Penetrating ulceration of the test into the perivisceral coelom sometimes occurred. Stars with any combination of the above clinical signs of disease (“lesions”) were used to characterize the pathogenesis of SSWD. Stars classified as lacking all gross clinical signs (“no lesions”) were placed in a holding trough outside at the GSO, which received flow-through ambient unfiltered water from Narragansett Bay (12–23°C depending on time of year, 2.9–3.3% salinity). Holding trough stars were monitored for at least 2–3 weeks for clinical signs of SSWD, and were fed every two weeks with snails or mussels collected from the GSO Pier. After an initial acclimation phase, stars lacking clinical signs were brought into the Pathology Laboratory in the Blount Aquaculture Research Building (GSO) and placed in 50 L glass aquaria containing 38 L of recirculating filtered (1 μm) and UV-sterilized (EU25-U, Pentair-Emperor Aquatics) seawater from Narragansett Bay (FSSW) or filtered artificial seawater (FASW) and maintained at temperatures ranging from 16–20°C, depending on the trial. Water was aerated through the use of air stones. Stars were monitored for an additional 2–3 weeks for signs of SSWD. If no clinical signs were observed, stars were used in experimental trials. An additional set of experimental stars was received from Charleston, South Carolina (SC) in April 2015 (n = 11). These stars were placed directly into 2 aquaria (n = 5, n = 6, respectively) containing 50 L of FASW at ambient conditions (19–23°C, 2.9–3.2% salinity) at the Blount Pathology Lab. For all experiments, water quality (total ammonia nitrogen and turbidity) was measured every 2–3 d, and water changes were performed as required to maintain water quality. Effluent water from all challenge experiments was treated with bleach and neutralized with sodium thiosulphate prior to disposal.

**Table 1:** List of collected sea star samples.

Site	# Collected	Sample Type	# Tested by qPCR
Beavertail, RI	17	Swab and Tissue	13
GSO Pier	37	Swab and Tissue	19
Maine State Aquarium	4	Swab and Tissue	3
Charleston, SC	11	Swab and Tissue	10

### Evaluation of disease range and timeline of epizootics

A questionnaire was designed to identify the location and extent of SSWD mortality events in the field and the water conditions associated with the die-offs. The questionnaire was distributed to 5 local dive groups (RI, Massachusetts—MA) and aquarists at the New England Aquarium (Boston, MA), Mystic Aquarium (Mystic, Connecticut—CT), and the Maritime Aquarium (Norwalk, CT). Samples of sea stars were received from several sites along the reported range (Table 1) and maintained as described in the section above. Samples of sea stars from these locations were collected under the institutional permits for the collecting institutions. Photographs, body condition scores, and swab samples were taken upon star arrival. The animals were observed daily for mortality and gross signs of disease. These signs were documented through photography using an Olympus S2X10, with LG-PS2 illuminator scope and Olympus DP72 camera. Tissue samples were collected from moribund and dead stars and processed as described below for histological examination (all stars) and for microbiological analysis (moribund stars only).

### Sample collection and processing

#### Sample collection

For each star (with and without clinical signs), two 1.5 mL microcentrifuge tubes were filled with 1 mL filtered artificial salt water (FASW, 2.8% salinity), labeled, and placed on ice. The animal to be sampled was placed in a sterile disposable Petri dish and rinsed three times with 10 mL FASW to remove surface debris. Photographs were taken as described above to document gross morphology of animal. Size, date, water quality from the holding tank, and body condition were recorded. Using a sterile swab, one 1 cm^2^ area of ray was swabbed gently, and the swab was rinsed into 1 mL of FASW. If stars showed signs of disease, a swab each was taken from lesions and from an area with no visible lesions. Tissue clippings (2–3 mm^3^) were collected from the aboral surface of lesioned and non-lesioned animals, and placed into microcentrifuge tubes with 1 mL of TRIzol (Sigma). Autotomized limbs and whole bodies were fixed in 10% formalin in seawater for histological examination. Swabs (after plating for bacteriological isolation, see below) were centrifuged for 10 min at 12,000 x g at room temperature. Once the supernatant was decanted, 1 mL of TRIzol fixative was added. Samples were stored in the -80°C until analysis.

#### Histological examination

Fixed tissues were decalcified in a 0.5 M EDTA/NaOH solution (pH = 8) for 48–96 h [14]. Cross and longitudinal sections of affected rays (and internal organs) and sections of central disk (and internal organs) were sampled from the prepared tissues. Hematoxylin and eosin (H&E) stained, paraffin embedded sections were prepared by Mass Histology Service (Worcester, MA, USA) using standard methods and were examined using an Olympus BX51 microscope fitted with a DP72 camera.

#### Bacterial culture, DNA isolation, and species identification

Swab samples were thoroughly mixed before preparing 3 serial 1/10 dilutions. An aliquot (20 μL) from each of 4 dilutions was plated onto Seawater Tryptone (SWT, prepared with FASW at 3.0% salinity), and Thiosulfate Citrus Bile Salt agar (TCBS) plates, incubated at room temperature (20–24°C), and monitored daily for bacterial colonies. Bacterial colonies in each of the media plates were classified based on morphology (color, shape, and type of growth) at 24 and 96 h after plating, and abundance of each colony type in colony forming units (CFU) per mL was recorded. Bacterial colonies enriched in diseased animals were resuspended in 5 mL SWT broth and grown for 36 h at room temperature with shaking. From this culture, 1 mL was placed in a tube with glycerol (20% volume) and stored at -80°C. Another 1 mL was pipetted into a clean 1.5 mL microcentrifuge tube and processed for DNA extraction and sequencing of 16S rDNA [15]. Sequences were compared to sequences in the Ribosomal DNA Database (RDP Release 11) for species identification.

#### DNA isolation for quantitative real time PCR

Sea star tissue and swab samples in 1 mL of TRIzol were removed from the -80°C freezer, placed in an ice bath to thaw, incubated at room temperature for 5 min, and homogenized using sterile RNA free pestles. DNA and RNA were isolated using the manufacturer’s protocol and stored at -80°C until use. The Thermo Scientific Nanodrop ND8000 version 2.2.1 system was used to determine DNA concentration.

### Bacterial challenge experiments

One of the potential bacterial pathogens (identified based on abundance and predominance in diseased sea stars and a species identity match suggestive of potential pathogenicity) was used in challenge experiments. Healthy-looking sea stars (5–200 g) were housed 2 to a tank (50 L) in 10 aquaria with FSSW for 7–10 d prior to experimentation at a temperature of 16–18°C, salinity 2.8–3.0%. Treatments (2 tanks per treatment) included: 1) Control (no pathogen exposure); 2) Animals immersed in seawater with 10^6^ CFU/mL of the candidate bacterium; 3) Animals immersed in seawater with 10^6^ CFU/mL of the bacterial isolate after cuticle abrasion (an area of 1 mm^2^ was eroded using sandpaper to induce cuticle loss to facilitate infection by breaking down the cuticle defenses [16]). 4) Animals injected with 0.1 mL of a 10^5^ CFU/mL solution of bacteria in FASW through the dorsal epithelium of one ray into the perivisceral coelom; 5) Animals injected with 0.1 mL FASW water (control). Stars were monitored twice daily for 10 d, and time to the appearance of clinical signs of SSWD (time to morbidity) and time to mortality recorded.

### Cohabitation challenge experiments

The purpose of the cohabitation trials was to assess transmission from diseased to healthy-looking stars and to assess the timeline of disease progression. Time to morbidity and mortality after the initiation of cohabitation were recorded, as well as changes in behavior and physical appearance of stars. Swabs of lesioned areas and tissue samples were collected for quantitative real time PCR analysis; the remaining tissues from selected animals were processed for histological analysis. All the experiments were performed in flow-through aquaria maintained as described in the Animal Husbandry section above. Records of water quality, temperature, and body condition of cohabitation stars were taken daily for at least 10 d or until all challenged stars were deceased.

#### Cohabitation with diseased Forbes sea stars

Four stars with signs of SSWD held in a trough at the GSO Aquarium Building (termed “Source”) were placed one to a tank into 4 tanks. Twelve healthy-looking acclimated *A*. *forbesi* (see animal husbandry section above; named “Challenged”) were placed n = 3 into each of the 4 aquaria with FSSW and allowed direct contact with diseased stars. Once the Source stars died, their bodies were removed from the tank and a 20% water change was performed every other day. Challenged stars were then monitored for signs of wasting for 10 d. An additional cohabitation trial was performed to collect infected water for immersion challenge experiments (see below).

#### Cohabitation with other diseased echinoderms

Diseased animals received from the Maine State Aquarium (one sea star *Asterias rubens*, one green sea urchin *Strongylocentrotus droebachiensis*, and 2 sea cucumbers *Cucumaria frondosa*) were received on April 1, 2014 and placed into each of 4 aquaria in FSSW (16–18°C, 2.9–3.1% salinity). Plastic mesh dividers were placed down the middle of the tanks to prevent direct contact between Source and Challenged animals. Three stars that had passed the acclimation phase and were negative for gross SSWD lesions were placed into each of the 4 tanks. Stars were monitored once daily for 3 weeks.

### Challenge experiments with infected and 0.22 μm-filtered infected water

The goal of these experiments was to determine if a virus/toxin is the causative agent of SSWD in *A*. *forbesi*. All these immersion experiments were performed in aquaria containing FASW to which at least 1 L of water from infected tanks (filtered or not) was added.

#### Collection and preparation of infected water

An additional cohabitation experiment was performed to test the effect of filtration in challenge experiments. This trial lasted for 40 d (October-November 2014), with consistent turnover of diseased stars. Source water (20 L, named “Infected water”) was taken from a tank in which SSWD had caused mortality and placed into a clean tank with 2 healthy-looking *A*. *forbesi*. Stars were monitored daily for signs of disease. When challenged stars began to show SSWD signs, swab samples were taken for analysis. Each time a star was determined moribund, the star was removed from the tank and processed for analysis, a 20% water change with FSSW was performed, and a new healthy-looking star was added to the tank. Water changes were also performed as needed to maintain water quality. A total of 15 stars were exposed in this way. Water from these tanks was used directly in the challenge experiments described below or collected in 500 mL bottles and stored at -80°C. Data on time to morbidity and mortality of stars was recorded and included in the “Cohabitation with diseased Forbes sea stars” analysis.

To prepare the water for challenge experiments, half of the water collected from infected tanks was filtered through a 0.22 μm filter (named “filtered water”) to remove bacterial or parasitic pathogens. Most viruses and toxins remain in water that passes through the filter [17,18]. For one of the experiments, and as an additional control, half of the volume of filtered infected water was treated with UV light (4–5 h contact time under SterilGARDHood VBM400, The Baker Company, Inc.).

#### Immersion challenge experiments using filtered infected water

Two filtration challenge experiments were performed, one with freshly collected infected water and one with frozen infected water. For experiment I, treatments included: A) freshly collected (named “fresh”) infected water; and B) 0.22μm filtered fresh infected water. Two healthy-looking *A*. *forbesi* were placed into each tank and monitored daily. The trial continued for 10 d. For filtration experiment II, infected water that had been previously frozen was used. Bottles containing frozen infected water were removed from the freezer and placed in an ice bath to thaw (20–30 min). Twenty-four healthy-looking stars were distributed into 8 aquaria (n = 3). Stars were immersed in antibiotics (Enrofloxacin 2.5 mg/kg) for one hour prior to treatment. Treatments, each performed in duplicate, included: 1) control, FASW only; 2) filtered and UV treated frozen infected water, 3) filtered frozen infected water, and 4) frozen infected water. Experimental stars were monitored for an additional 3 weeks for signs of SSWD. Swab samples were collected for processing before stars entered treatment, when they started to show clinical signs, and at death.

### Amplification, cloning and sequencing of SSaDV DNA from *A*. *forbesi*

Polymerase Chain Reactions using transcript specific primers designed to the sequence of the SSaDV VP1 (accession no. PRJNA253121; FWD: 5'-ACGAAGATCCTGTGGTGAGTT-3'; REV: 5'- CATCGGTGTACAATATCCTGCTA-3') and VP 4 (FWD: 5'-GGAATCTTGCTGATGAAAC AGC-3'; REV: 5'-GAGCTGCTGATTTTGTTCAGG-3') genes, were carried out on isolated DNA from samples of *A*. *forbesi* with and without clinical signs of SSWD. Samples were tested in duplicate. For each sample, a volume of 37.3 μL of Platinum Taq Master mix (Qiagen) was added to each sample tube containing 2.5 μL of DNA template, along with 5 μL of each primer (stock concentration = 10 μM), and 0.2 μL Platinum Taq. Samples were run through a Mastercycler^®^ Nexus X2 (Eppendorf) with the following cycling conditions: 94°C for 10 min, then 35 cycles of 94°C for 30 s, 50°C for 30 s, 72°C for 90 s, with a final extension at 72°C for 5 min. Amplicons were run on a 1% agarose gel in TAE buffer (40 mM Tris, pH = 7.6, 20 mM acetic acid, 1 mM EDTA) for 30 min at 100 V, stained, destained and bands were visualized using the BioRad Quantity 1 System. Bands corresponding to the predicted amplicon size for VP1 and VP4 (285 and 942 bp, respectively) were cut using a sterile razor blade, and placed into individual 50 μL microcentrifuge tubes. Amplicon DNA was purified from the gel and cloned using the pGEM-T Easy Vector System 1 method (Promega) following the manufacturer’s protocol. Plasmid DNA was isolated using a QiaPrep Spin MiniPrep kit and DNA concentration (μg/μL) was quantified using the Thermo Scientific Nanodrop ND8000 version 2.2.1 system. Samples were prepared for sequencing following standard procedure for the RI Genomics and Sequencing Center at the University of Rhode Island [http://web.uri.edu/gsc/].

### Quantification of SSaDV in *A*. *forbesi* using quantitative real time PCR

A Taq based assay for quantitative real time PCR for detection of the VP1 protein of SSaDV was used to quantify viral DNA. Primers and probe for quantitative real time PCR (FWD: 5’-GAC GTG CAA GAA GCT GAT AGA-3’; REV: 5’GTC CAA TAT AAC CAG CAA TAG AAT GAG-3’; PRB: 5’-ACG TCA GTA AGA AAT TCA CCA GCC C-3’) were developed using a consensus sequence derived from a ClustalW alignment (SDSC Biology WorkBench) of 3 sequences obtained from *A*. *forbesi* samples (see above) and a sequence provided by I. Hewson for VP1. Quantitative PCR was run in 20 μL reactions containing 1X Probes Master (Roche), 200 nM of each primer, 250 nM of probe, and 1 μL of isolated DNA per single reaction. A Roche LightCycler480 Real-time PCR Instrument was used to perform thermal cycling. The program consisted of a 5 min denaturing step at 95°C, followed by 60 cycles of denaturing and annealing (95°C for 30 s, 55.5°C for 30 s, respectively). Dilutions (ten-fold over six orders of magnitude) of a plasmid containing the VP1 target region were used as a standard to estimate VP1 concentration. A positive control (sample containing SSaDV DNA) was kindly provided by I. Hewson (Cornell University). Plasmid DNA containing *A*. *forbesi* VP1 (cloned as described above) was used to standardize target viral DNA results based on the number of VP1 DNA copies per μL of sample.

### Data analysis and statistics

Statistical analysis and figures were generated using Prism 6 for MacOS (GraphPad Software Inc.). Mortality data from the challenge experiments was analyzed using a Keplen Meir survival analysis. Average time to mortality and morbidity was analyzed using a Kruskal-Wallis non-parametrix test. Data on SSaDV VP1 copy number per sample was analyzed using one-way ANOVA followed by Tukey’s post-hoc tests or Student t-test (when 2 treatments were compared) on log10-transformed data. Significance was established at a level of p<0.05.

## Results

### Timeline and range of the SSWD epizootic on the Atlantic Coast of North America

**Fig 1:**
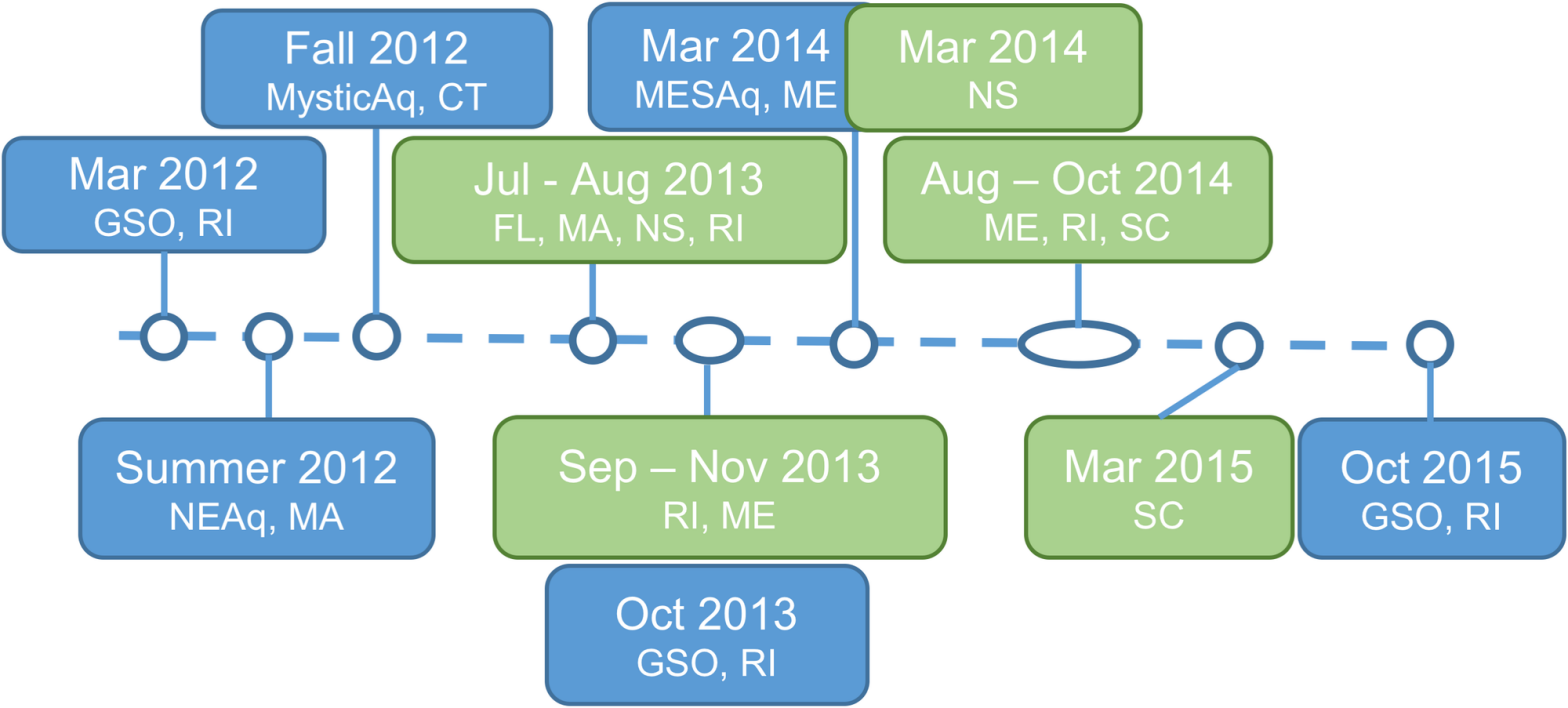
Timeline of SSWD events in sea stars on the Atlantic Coast of the USA. The timeline summarizes reports of events observed in aquariums (in blue) and citizen reports (green) in response to questionnaires administered in 2013 to 2015.

**Fig 2:**
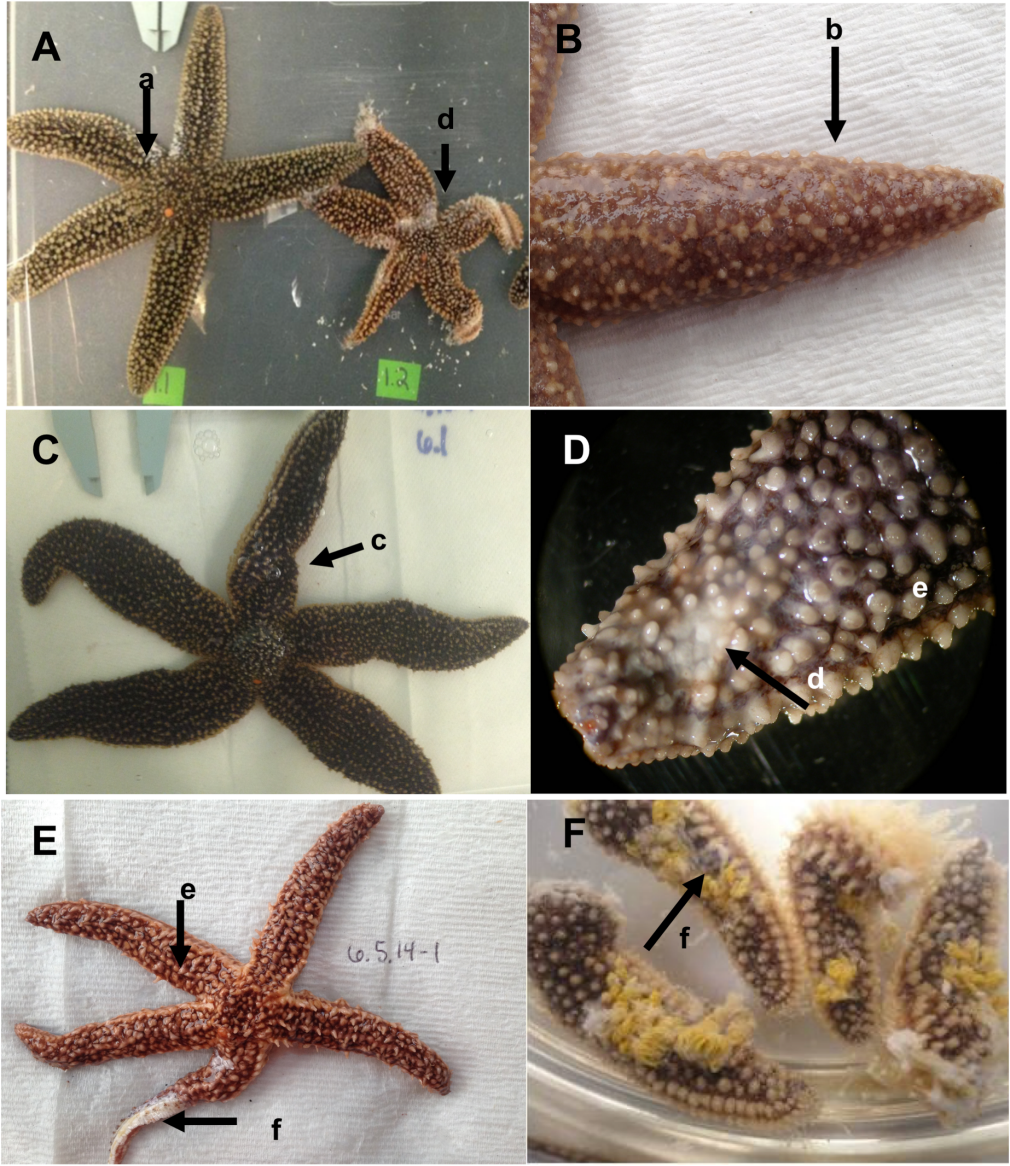
Gross morphological signs of Sea Star Wasting Disease. Representative images of *A*. *forbesi* affected with wasting disease showing combinations of different clinical signs (A-F). Clinical signs are indicated by arrows, and included (a) limb curling, (b) mucoid-like aboral surface coat, (c) pinching, (d) small ulcerations shown as white pinpoint lesions, (e) loss of spine orientation, and (f) severe ulcerations leading to exposure of underlying tissues.

An approximate timeline was established for the SSWD Atlantic Coast (USA and Canada) outbreak based on our direct observations and citizen and aquarium reports from questionnaires (Fig 1). Prior to summer 2011, populations of *A*. *forbesi* in Narragansett Bay, RI, were abundant, based on our direct observations of collections in oyster bags during routine disease monitoring. Our first direct observation of SSWD was in March 2012, when 10 *A*. *forbesi* were brought into a holding tank at the GSO Aquarium Building from the GSO Pier for an unrelated experiment. Within 5 d, all stars showed signs of wasting, including loss of turgor pressure, curled limbs, and lesions that lead to ulceration of the aboral surface and death (Fig 2). All stars perished within a week of placing them in the tank. Two major die-off events in *A*. *forbesi* maintained in our holding troughs were observed in October 2013 and 2014, both at periods associated with rapid temperature decreases in Narragansett Bay (~0.3°C per d). During the summer and fall of 2012, observations of sea stars with gross clinical signs of SSWD were reported by questionnaire responders in aquaria held stars at the New England Aquarium, Boston, MA and the Mystic Aquarium, Mystic, CT. Reported cases at the New England Aquarium were limited to *A*. *forbesi* and *A*. *rubens* collected from Cape Cod and brought into holding tanks. At the Mystic Aquarium, both *A*. *forbesi* and the sunflower star, *Pycnopodia helianthoides*, showed signs of wasting. Samples from Mystic were sent to the Hewson and Breitbart labs for analysis (Tuttle, personal communication; [8,12]). In March 2014, echinoderms collected from the field and placed in the touch tank at the Maine State Aquarium quickly showed signs of lethargy, loss of turgor pressure, and lesion development. These echinoderms had experienced temperature stress during transport to the aquarium (Ayden-Rodrigues, personal communication). These clinical signs were also seen in echinoderms cohabitating with the diseased specimens introduced in the tank. Specimens of cohabitating echinoderms showing signs of disease were sent to URI for analysis (Table 1). Other public aquariums reporting signs of disease on Atlantic and Pacific Coast stars in display/touch tanks include the National Aquarium, Baltimore, Maryland (2014).

In 2014–2015, we received several citizen reports of wasting observed in field populations of sea stars from Nova Scotia to Florida (Fig 1 and S1 Table). In Rhode Island, diseased *A*. *forbesi* and *A*. *rubens* were observed in at the Graduate School of Oceanography (Narragansett, RI; URI Dive Group 2014, personal communication), Fort Adams State Park (Newport, RI), Great Salt Pond (Narragansett, RI), and King’s Beach (Newport, RI; Kalipso Dive Group 2013–2014, personal communication). North of RI, we received reports from Marblehead, MA (Lebowitz 2013, personal communication), Stonington, Northport, and Lincolnville, Maine (McDonald 2013 and Sprague 2014, personal communication) and Dalhousie University and St. Margaret’s Bay, Nova Scotia, Canada (Scheibling 2015, personal communication). South of RI, wasting in *A*. *forbesi* was reported in Hilton Head, South Carolina (SC; Mahoney 2014, personal communication) and along the Coast of Charleston (Boylan 2015, personal communication). Morbidity (loss of turgor, lesions, disintegration) was reported in cushion stars, *Asteroidea oreaster reticulates*, along the Coast of Florida (Lureen 2013 personal communication). A full list of reports can be found in S1 Table.

### Clinical signs in field and aquarium-collected echinoderms

Two different pathologies were observed in *A*. *forbesi* collected from the Atlantic Coast of the USA and those stars kept in our holding systems: an acute form (similar to that reported in Pacific Coast epizootics; [8], leading to death within 7–10 of the start of clinical signs, and one that was chronic and progressed more slowly. Signs of the acute form included loss of body turgor (stars showed a deflated appearance and lack of overall rigidity; Fig 2A) and a mucoid-like surface change that caused the spines to appear smooth and glossy (Fig 2B–2D). A pinched or puffy appearance was common, in which a compression on a focal point occurred along or at the base of the rays (Fig 2C). More severe signs included the presence of white lesions along the arms, which started as pinpoint lesions of soft tissue (Fig 2A–2D) and progressed in most stars to large areas of exposed underlying white ossicles (Fig 2E and 2F). Limb autotomy (“dropping” of the limbs, Fig 2F) was also observed in many stars. The chronic form showed a much slower progression. Stars exhibited lethargy (defined as an observable decrease in activity level and righting response time) with the development of small pinpoint (<3 mm^2^) lesions (similar to those in Fig 2B). These lesions could persist for weeks to months before any mortality was noted, if at all. A limited number of animals (n = 5) exhibited this form, as these stars survived with minor lesions for 115 ± 74 d.

### Histological examination of field and aquarium-collected *A*. *forbesi*

**Fig 3:**
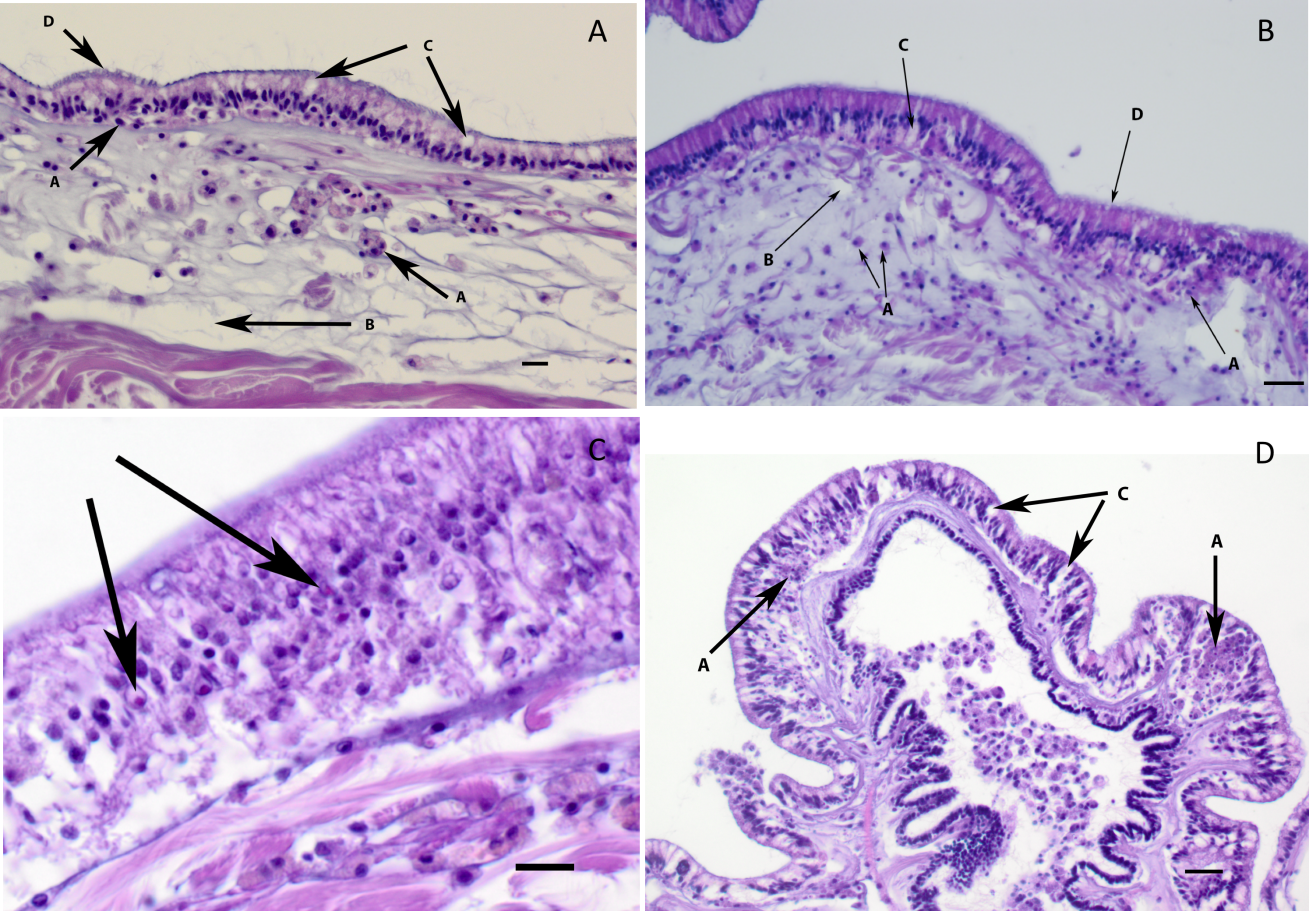
Histological examination of tissues from Forbes Sea stars with SSWD. Representative images with examples of the most characteristic morphological changes to tissues and cells associated with SSWD, as indicated by arrows. (A) and (B): Early stages of infection characterized by an influx of hemocytes (a) in the dermis and in the epithelium underlying or between epithelial cells, oedema of the tissues (b) and early intra- and intercellular vacuolation of the columnar epithelium and cuticular disruption (d). (C) Larger magnification of an area in the epidermis showing pink round to irregular inclusions that were sometimes noted in the vacuolated nuclei of affected epithelial cells (thicker arrows). (D) Moderate to severe changes in the epithelium and dermis of this papilla characterized by intra-epithelial accumulation of necrotic and intact hemocytes (a), intra and inter-cellular vacuolation of the columnar epithelial cells (c) and dermal oedema (6 μm, H&E stained paraffin section; bar = 30 μm).

All stars examined for histology (n = 22) showed gross morphological signs of disease, but with different levels of severity. Stars with gross signs of SSWD exhibited mild to severe necrosis of epidermal tissues (Fig 3) often resulting in ulceration of the aboral epithelium and exposure of the underlying ossicles and associated connective tissues (resulting in white lesions grossly). Lesions were characterized by cuticle disruption and loss associated with intra and intercellular vacuolation of columnar epithelial cells of the epidermis (Fig 3A, 3B and 3D). In early lesions, often mild influxes of amoebocytes (hemocytes) were noted (Fig 3A–3C). In ulcerated lesions, the epithelium was lost and the underlying dermal connective tissue was oedematous and/or necrotic. Amoebocytes varied in number from few (Fig 3A) to many in connective tissues and ossicles underlying the ulcer (Fig 3D). In ulcerated areas, the ossicles appeared to have degenerated with collapse of the net-like appearance of the supporting connective tissues. This collapse was more severe and frequent than the collapse sometimes seen in normal samples due to decalcification, which removes the mineral from the ossicles leaving only the thin trabeculae of supporting connective tissue in histological sections. The presence of small numbers of ciliates and bacteria were noted in the dermis of some samples, but were not consistently tied to lesion presence. Small, 1 μm eosinophilic inclusions in some affected columnar epithelial cells were noted in some samples (n = 2), but were not seen consistently identified in diseased tissues.

### Quantification, isolation and characterization of bacteria from Forbes stars with SSWD

**Fig 4:**
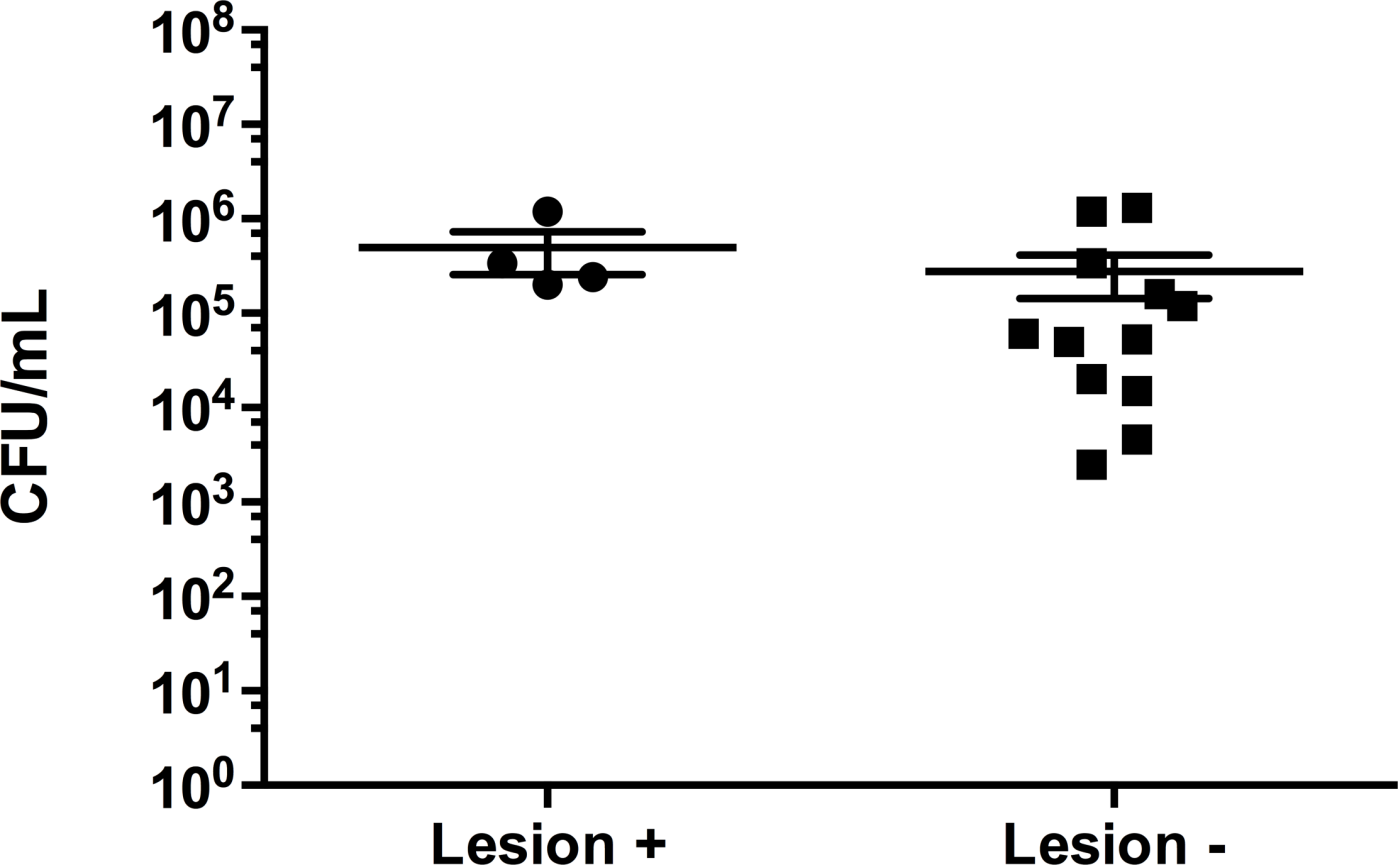
Average colony forming units (CFU/mL) of bacteria from Forbes stars with or without signs of SSWD. Culturable bacteria (individual sample values and average CFU/mL ± SEM) from swabs obtained from stars with visible SSWD lesions (lesion +) and those with no visible lesions (lesion -).

On average, stars that did not show lesions of SSWD had a lower amount of bacteria in CFU/mL of swab sample, though it was not statistically significant (p > 0.05; Fig 4).

The bacterial morphotype showing a relatively higher abundance in stars with lesions as compared to healthy-looking stars was collected from the plates and identified through sequencing of 16S rDNA. Colonies were raised and circular with an off-white/light yellow coloration, and the margins were smooth to undulating in shape. Sequences from this bacterial isolate showed the highest levels of sequence identity to a *Roseobacter* sp. (Accession no: JX53058). This isolate was selected for the bacterial challenge experiment.

**Fig 5:**
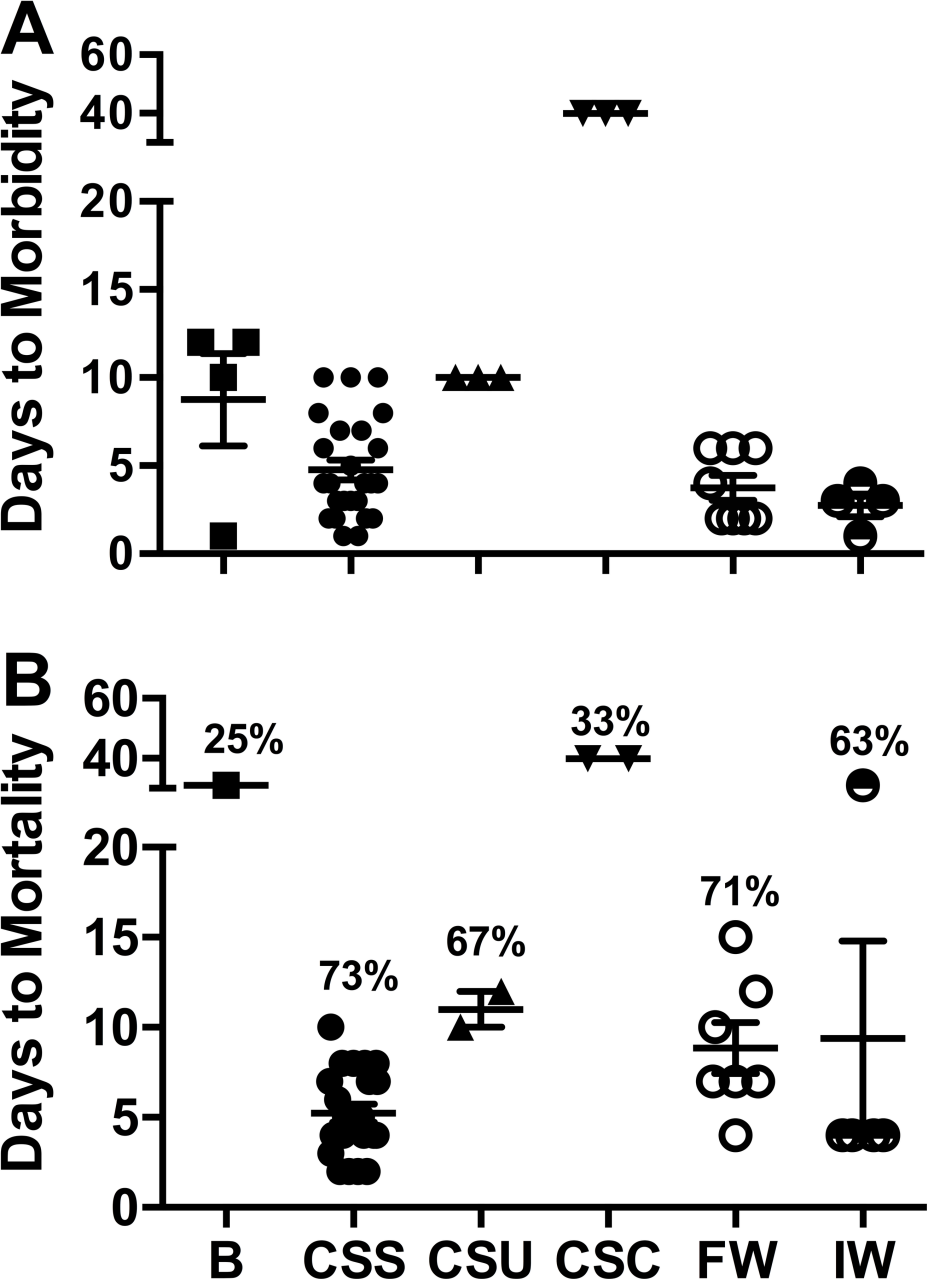
Time to morbidity and mortality in experimentally challenged Forbes sea stars. Time in days to observation of the first clinical signs of SSWD (A) and mortality (B) in challenged stars. Each symbol represents an individual star. Plots also show the average ± SEM (horizontal and vertical lines respectively). The cumulative percent mortality in each treatment is also shown in (B). Treatments (on the x axis) were: B = Immersion challenge with a *Roseobacter* sp. Isolate (n = 4); CSS = Cohabitation of healthy-looking *A*. *forbesi* with stars with signs of SSWD (all experiments combined, n = 30); CSU = Cohabitation of healthy-looking Forbes sea stars (n = 3) with a sea urchin from the Maine Aquarium; CSC = Cohabitation of sea stars (n = 6) with 2 sea cucumbers from the Maine Aquarium; FW = Immersion in filtered (0.22 μm) infected water (n = 8); IW = Immersion in infected water (n = 8).

Stars (*A*. *forbesi*) exposed by immersion to the *Roseobacter* sp. isolated from lesions of SSWD stars exhibited lethargy, weak tube feet attachment to substrate, and slow righting response within 9 d of being exposed to the bacteria and 25% cumulative mortality (1 star dying on day 31 after challenge; Fig 5), while control stars did not show any signs of morbidity or mortality. Mortality was also seen in 3 sea stars (25% cumulative percent mortality) injected with FASW (control for bacterial injection), which may have been due to stress caused by handling. None of the animals used in this experiment exhibited the loss of turgor pressure, limb curling, or lesion development characteristic of SSWD. We were unable to re-isolate the *Roseobacter* sp. from affected stars. These results indicate that this bacterium is not the causative agent of SSWD in sea stars, based on differences in lesion presence and time to morbidity and mortality.

### Cohabitation challenge experiments

#### Cohabitation with diseased Forbes sea stars

In the 3 cohabitation trials performed with diseased *A*. *forbesi* (including the sea star received from the Maine State Aquarium), most (83%) healthy looking *A*. *forbesi* started showing the characteristic signs of SSWD starting 3 d after placing a moribund *A*. *forbesi* into the tank, with 73% of the exposed stars dying within 10 d after the start of the cohabitation (Fig 5). No mortality was observed in control stars.

#### Cohabitation with other diseased echinoderms

Preliminary cohabitation challenge experiments in which *A*. *forbesi* affected by SSWD cohabitated with apparently healthy *P*. *miniata* from the Pacific Coast showed that transmission of SSWD can occur between *A*. *forbesi* and a Pacific Coast species. Observations from a disease outbreak at the Maine State Aquarium, during which several echinoderm species, including *A*. *forbesi*, sea urchins (*Stronglyocentrotus droebachiensis)*, and sea cucumbers (*Cucumaria frondosa*) showed signs of disease, suggested that echinoderms other than sea stars may also be affected by SSWD. In order to determine if the disease occurring in these echinoderms had the same etiology as SSWD, we performed a cohabitation challenge experiment in which healthy looking Forbes sea stars were placed with diseased echinoderms from the Maine State Aquarium. Of the 3 *A*. *forbesi* cohabitating with the diseased sea star included in the specimens received from the Maine, 1 showed signs of SSWD and died by day 10 (Fig 5, included in the CSS group). The 3 *A*. *forbesi* cohabitating with the diseased sea urchin showed the characteristic signs of SSWD by day 10 post-exposure, and 2 died by day 12 (Fig 5). Of the 3 *A*. *forbesi* exposed to one of the diseased sea cucumbers, 3 showed signs of disease starting on day 40, and 2 died shortly after signs were observed. Signs of disease in these stars were similar to those observed in bacterial challenges, and were not consistent with SSWD. No signs of disease or mortality were observed in the 3 stars cohabitating with the other diseased sea cucumber or in control tanks.

### Immersion challenge experiments using filtered and unfiltered infected water

In the 2 immersion challenge experiments that were performed, several (5 of 8) of the *A*. *forbesi* exposed to unfiltered infected water showed signs of SSWD by day 3 post-immersion, reaching a cumulative percent mortality of 25% (one star died on day 32 and the others on day 3) in one experiment and 100% in the other (Fig 5). All *A*. *forbesi* exposed by immersion in 0.22-micron filtered infected water showed signs of wasting 2 to 6 d post-exposure, reaching an average of 71 ± 6% (SD) cumulative mortality by day 15. No morbidity or mortality was observed in control tanks.

### Detection and cloning of SSaDV VP1 in *A*. *forbesi*

Several samples selected for end-point PCR testing showed amplification using primers designed from the VP1 and VP4 sequences of SSaDV. Bands matching the expected range of VP1 and VP4 (285 and 492 bp, respectively) were selected for cloning and sequencing. Sequencing of the cloned fragments amplified with the VP1 primers showed identity to the SSaDV VP1 sequence in GenBank (Accession KY785180, KY785181). However, none of the cloned inserts amplified with the VP4 primers matched SSaDV sequences (S1 Text).

### Concentration of SSaDV VP1 gene in Forbes sea star samples

Of the 48 stars tested in the qPCR, 29 (60%) tested positive for the presence of the SSaDV VP1 gene (S2 Table). Samples of DNA of *A*. *forbesi* showed Cp values ranging from 8.9 (corresponding to 2.1 x 10^11^ copies of VP1 per μL of sample, based on a standard curve using the cloned VP1 target) to 37 (negative cut-off), with an average (±SD) Cp of 28.1 ± 9.8 (3.1 ± 6.2) x 10^10^ copies/μL). The positive control provided by Hewson had a Cp of 33.6 ± 3.8 (corresponding to (6.8 ± 9.7) x 10^5^ copies VP1/μL). Two types of samples were collected from stars: a skin swab (swab resuspended in 1000 μL) and a tissue sample (approximate weight = 0.2 mg). Copy number of the SSaDV VP1 gene in a skin swab samples averaged (1.9 ± 0.8) x 10^10^ copies/μL, while tissue samples averaged (1.8 ± 1.5) x 10^10^ copies/ μL. No significance difference (p = 0.7) was observed in copy number or % of positive samples between swabs and tissue samples, so data from either source was included in further analysis (S2 Table).

**Fig 6:**
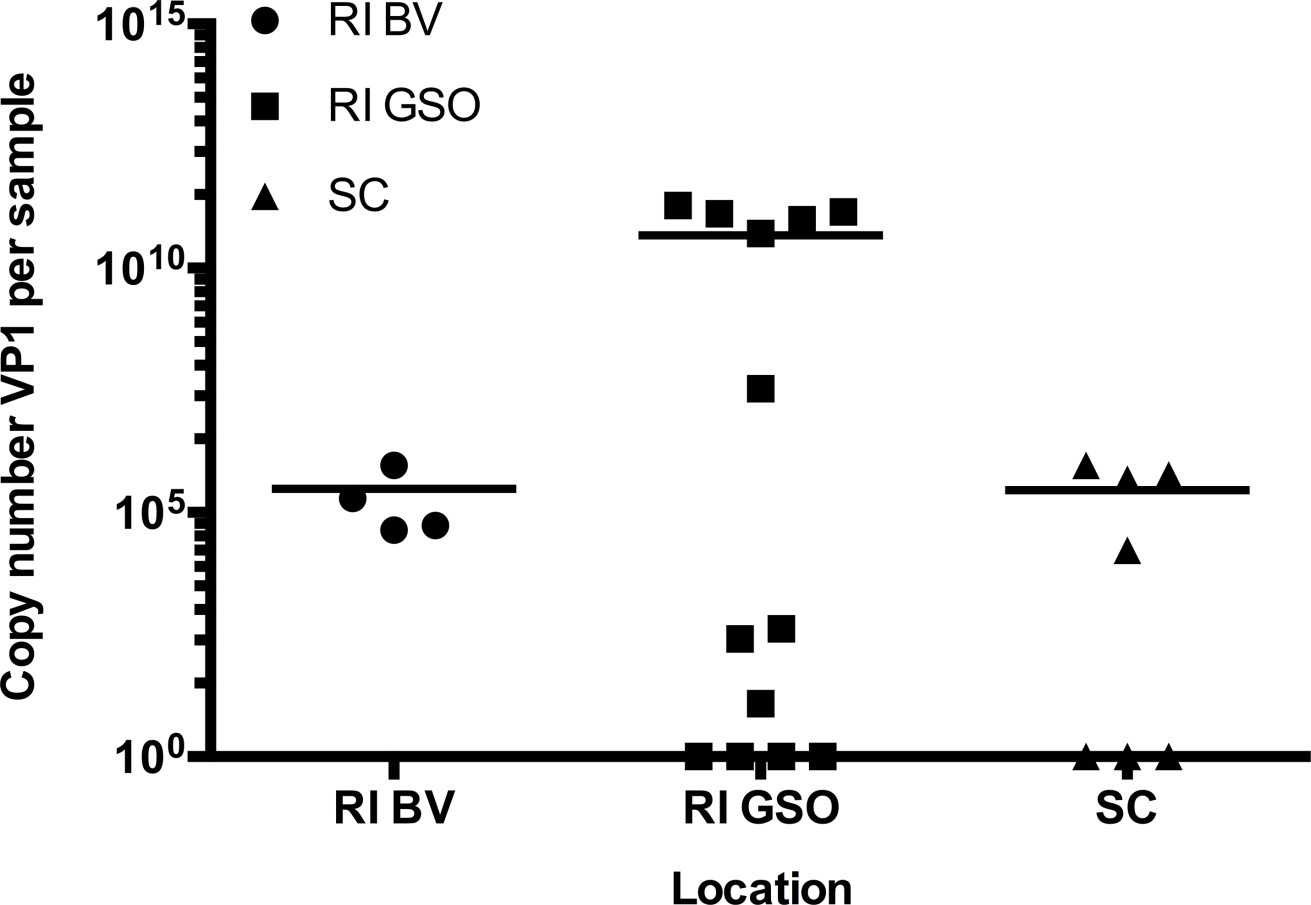
SSaDV VP1 copy number in field Forbes sea stars from 3 locations on the Atlantic Coast of the USA. Concentration of SSaDV VP1 (copy number/μL) in all sea stars collected in different locations from the Atlantic Coast of the USA between 2013 and 2015. Plot shows individual values for each sample (symbols) as well as the mean (line). RI BV: Beavertail, RI; RI GSO: Pier at the Graduate School of Oceanography, RI; SC: Charleston, SC (3 d after arrival).

Of the 34 field-collected samples tested, 14 were positive for VP1 (41%), with a concentration (mean ± SD) of (3.0 ± 7.0) x 10^10^ copies/μL (Fig 6). The highest levels of SSaDV VP1 were detected in samples from the GSO pier in Rhode Island. According to location, prevalence of SSaDV was 100% (4/4 samples) in Beavertail, RI, 60% (9/15) at the GSO pier (RI), and 38% (6/16) in SC. A significant difference in VP1 copy number was seen between SC and GSO samples (p<0.05, One-Way ANOVA and Tukey’s post-hoc test).

**Fig 7:**
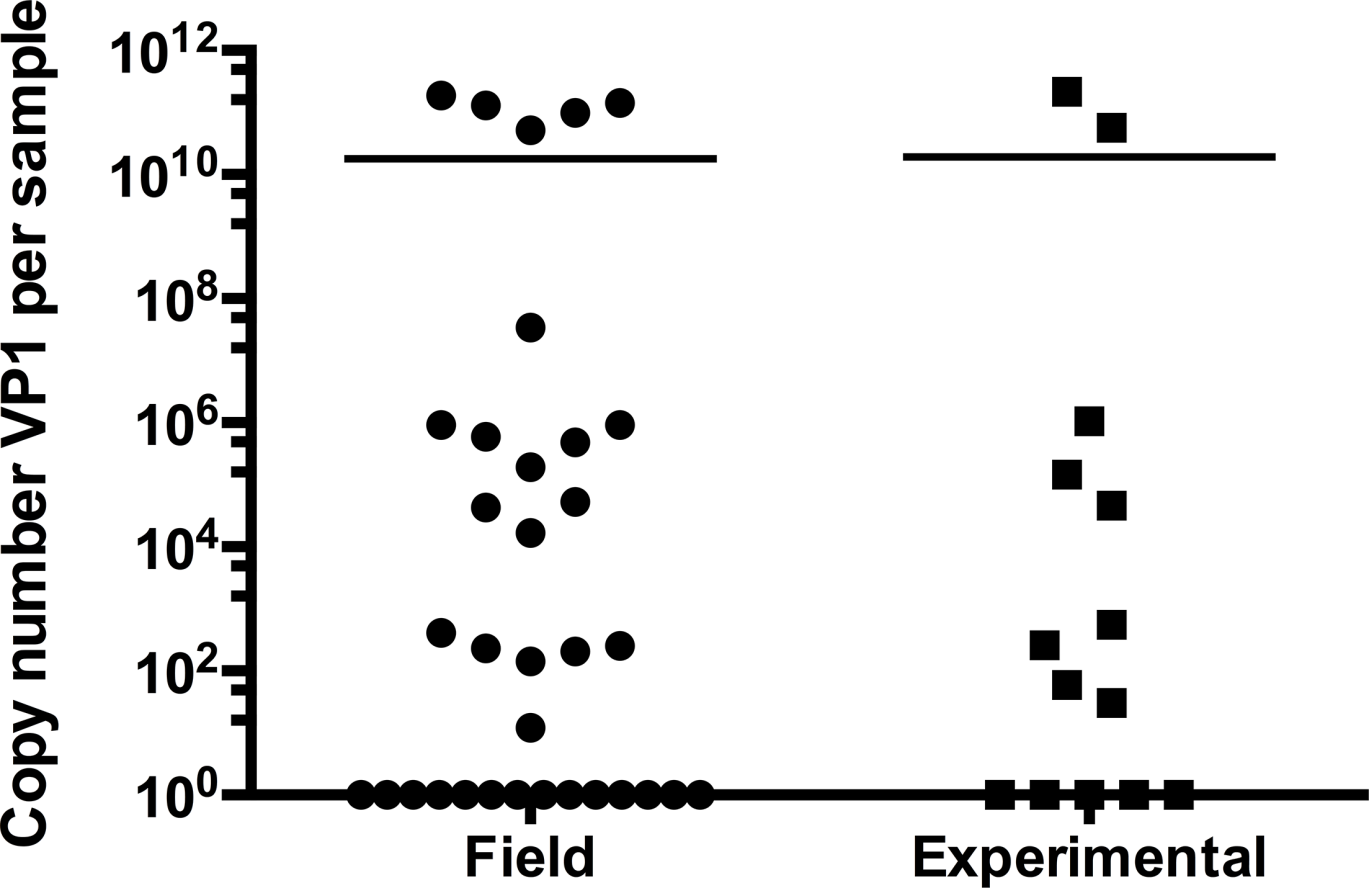
SSaDV VP1 copy number in field collected and experimentally challenged stars. Comparison of the concentration of SSaDV VP1 (copy number/μL) in *A*. *forbesi* collected from the field (n = 34) and from experimental challenges (n = 14). Plots represent individual values for each sample (symbols) and the mean (line).

Of the 14 sea stars from experimental challenges sampled for determination of SSaDV load, 9 were positive for VP1 (64%), with an average concentration of (3.0 ± 7.0) x 10^10^ copiesVP1/μL of sample (Fig 7). VP1 DNA concentration was not statistically significant (p = 0.462, Student t test) between field-collected and experimentally challenged samples.

There was no significant difference in VP1 DNA copy number between stars showing signs of SSWD and those not showing signs of disease (p = 0.478; S2 Table). Of all the samples analyzed in this study (field and experimental), 20/48 (42%) of the samples that tested positive for the VP1 gene showed clinical signs of SSWD (Table 2). Additionally, 5/48 (10%) of the samples testing negative for the VP1 gene were also negative for clinical signs. A total of 23/48 (48%) of the samples show no concordance between the 2 tests, either testing positive for the VP1 region, yet showing no clinical signs, or testing negative and showing clinical signs. Contingency analysis using Fisher’s exact test yields a p-value of 1.0, suggesting that there is no statistically significant relationship between presence of clinical signs of SSWD and presence of VP1 DNA in this samples. Concentration of VP1 DNA in VP1-positive stars with no lesions ranged from 4.7 x10^2^ to 1.2 x10^11^ copies/μL.

**Table 2:** Relationship between clinical signs of SSWD and presence of SSaDV VP1 DNA. Contingency table expressing relationship between presence or absence gross morphological (clinical) signs and detection of SSaDV VP1 DNA using qPCR. Samples tested include a mix of samples from field-collected (n = 34) and experimentally challenged *A*. *forbesi* (n = 19). No relationship was observed between presence of VP1 and clinical signs of SSWD (Fisher’s exact test: p = 1.0).

	+ Gross	- Gross	Totals
+ qPCR	20 (42%)	9 (19%)	**29 (60%)**
- qPCR	14 (29%)	5 (10%)	**19 (40%)**
**Totals**	**34 (71%)**	**14 (29%)**	**48**

**Fig 8:**
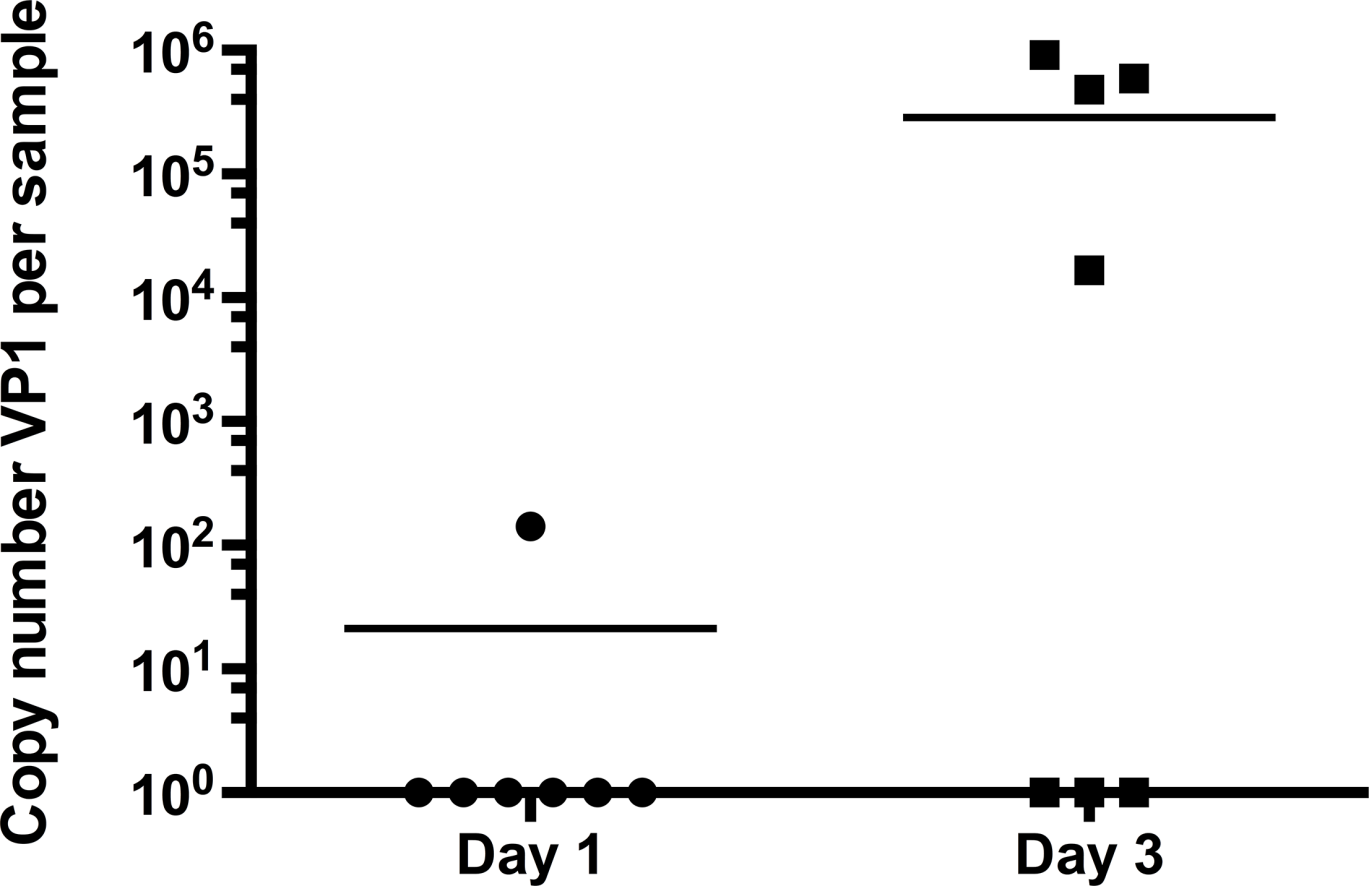
Change in SSaDV VP1 copy number in sea stars during progression of SSWD. Comparison of VP1 copy number per sample in sea stars received from the field in Chaleston, SC. Between the day of arrival (day 1) and day 3 after placement of the stars in quarantine/isolated tanks containing filtered UV-treated artifical sea water. Plots represent individual values for each sample (symbols) as well as the mean (line).

We sought to determine if viral load would increase in moribund stars as the disease progressed. For stars received from Charleston, SC, we were able to collect samples from the same sea stars at different stages of the disease for quantification of VP1 DNA. On the day of arrival from SC, 1/7 sea star was positive for VP1 DNA, with 142 copies/μL of sample (Fig 8). This star showed no signs of SSWD. At day 3 post-arrival, stars had experienced 100% mortality, while showing the characteristic signs of SSWD. Concentrations of VP1 DNA had significantly increased in 3 of the 6 sea stars to an average (±SD) of (2.7 ±3.8) x 10^6^ VP1 copies/μL of sample (Paired t-test on log-transformed data, p = 0.0138).

## Discussion

In this study, we sought to characterize SSWD in sea stars (*A*. *forbesi*, *A*. *rubens)* collected from the Atlantic Coast of the USA, determine if a virus could be causing this disease through experimental challenges, and assess the presence and relationship of SSaDV to clinical signs of SSWD in *A*. *forbesi*. Our results show that: 1) signs of SSWD have been reported in populations of *A*. *forbesi* and other sea star species from Florida to Canada in the period from 2012–2015; 2) Clinical signs of SSWD in *A*. *forbesi* from the Atlantic Coast of the USA are similar to those seen in sea stars on the Pacific Coast of the USA; 3) A viral pathogen is the most likely cause of SSWD in *A*. *forbesi;* and 4) Although levels of SSaDV increased with progression of the disease in one experiment, we did not observe a clear association between the presence of SSaDV in sea stars and gross clinical signs of SSWD.

Clinical signs and a timeline of disease progression have been defined here for *A*. *forbesi* based on gross observations and histological examination. Until now, very little was known about sea star wasting on the Atlantic Coast of the USA. We observed two different manifestations of the disease in *Asterias* spp.: an acute and a chronic form. The acute manifestation of the disease shows signs and times to morbidity and mortality that are similar to those described for sea stars affected by SSWD on the Pacific Coast of the USA [8,10,11,19]. The chronic manifestation (observed in only a handful of stars) may present itself with clinical signs early (3–5 d post exposure), but then linger for weeks to months before progressing rapidly to deterioration of sea star tissue. The two manifestations may be a reflection of different modes of progression of the same disease due, for example, to differences in host susceptibility, environmental conditions, or the impact of secondary and possibly tertiary pathogens. Recent work suggests that there is differential susceptibility and progression to death between juveniles and adults, which may be altered by changes in environmental conditions [9,10,19]. Alternatively, this more chronic form may be a manifestation of diseases caused by a different pathogen. Results from our bacterial challenges, in which exposed sea stars showed a slower progression of the disease and lower mortality, support this alternative explanation, suggesting that the chronic signs of disease we observed in some of the sea stars in our experiments were not associated with SSWD. Future work should seek to assess the relationship of the two manifestations of SSWD in *Asterias* spp. and examine the differential susceptibilities among different species, age ranges, and sizes, as well as geographical location.

Historical reports of sea star wasting outbreaks along the Pacific Coast of the USA have been loosely tied to increased water temperatures serving perhaps as an influx of pathogens, and/or as an additional stress factor on a subclinical diseased population [7,9,19]. Though some studies from the Pacific Coast have linked increased temperature to increased disease prevalence, it is unclear whether the temperature is a driving force, or if the stress induced by the rapid change is impacting the susceptibility to or virulence of SSWD [5,9,10,19]. The two major die-off events observed in the holding troughs at GSO were both associated with rapid, seasonal, temperature decreases in Narragansett Bay, suggesting that changing temperature conditions may be a trigger for disease epizootics. A monitoring program of field populations on the Atlantic Coast of the USA, similar to the monitoring program in the Pacific Coast, is needed to examine the relationship of mortality events to potential triggers of the disease, including changes in environmental conditions such as temperature, salinity, and pH, as well as potential relationships with decadal fluctuations in climate and food availability [20–22].

Results from our cohabitation experiments suggest that the disease is highly transmissible in *A*. *forbesi*, leading to rapid and severe morbidity and mortality within 10 d of initial exposure. Furthermore, filtration trials involving water collected from tanks with diseased sea stars indicate that a viral pathogen is the most likely cause of SSWD in *Asterias* spp. on the Atlantic Coast, based on the similarity in patterns of morbidity and mortality. A viral pathology is also consistent with the conclusions by Hewson et al. [8] for the SSWD outbreaks on the Pacific Coast of the USA. Although several bacterial species have been found to be pathogenic to echinoderms [23] and some morbidity and mortality was observed in *A*. *forbesi* exposed to bacteria isolated from SSWD stars, the time to morbidity and mortality (significantly longer for stars exposed to bacterial challenge) and the gross and histological signs of disease observed in stars from the bacterial challenge are not consistent with SSWD.

Our research also explored the possibility that species of echinoderms other than sea stars could be susceptible to SSWD. Results of a small scale experiment in which healthy-looking Forbes sea stars were placed in tanks with one sea urchin, *Stronglyocentrotus droebachiensis*, and two sea cucumbers, *Cucumaria frondosa*, showing signs of disease indicate that the sea urchin may have been affected with SSWD disease. This conclusion is based on the fact that cohabitation with the sea urchin led to transmission to Forbes sea stars of a condition with gross signs, time to morbidity and mortality, and percent cumulative mortality consistent with SSWD. The results from the sea cucumber cohabitation experiments, however, were inconclusive. Differences in patterns of morbidity and mortality in the cohabitation experiments between the 2 sea cucumbers, and between the sea cucumbers and the sea stars used as source, suggest that the sea cucumbers may not be susceptible to SSWD.

Hewson et al. (2014) [8] identified a densovirus that has been associated with SSWD in species along the Pacific Coast of the USA. The research team identified three gene sequences (VP1, VP4, NS1) that code for parts of the Sea Star associated Densovirus (SSaDV) genome. The VP4 and NS1 sequences were used primarily for analysis in Pacific Coast samples, but we were not able to recover these sequences in our samples, perhaps due to differences in sequences between the viruses from different locations. We therefore relied on the sequences obtained for the VP1 gene to quantify viral DNA in *Asterias* spp. A majority of the samples tested (61%) have tested positive for the SSaDV VP1 gene, showing that Atlantic Coast stars do in fact carry the SSaDV VP1 DNA sequence, though at varying concentrations. Although the few stars (3 out of 6) in which we were able to quantify viral DNA at progressive stages of the disease show a clear increase in viral DNA copy number, our comparisons of VP1 DNA prevalence and gross signs of SSWD in samples randomly selected from our experiments do not provide conclusive evidence that presence of SSaDV is associated with SSWD in *A*. *forbesi*. Our results, restricted to quantification of VP1 DNA, should also be tested through analysis of additional targets in the sequence of SSaDV. Sequencing and characterization of SSaDV from *Asterias* spp. would allow for the development of other screening tools for SSaDV.

Our research strongly suggests that swab samples (a non-lethal collection method) could be used to screen samples for presence or absence of SSaDV in sea stars from the Atlantic Coast of the USA. DNA isolated from sea star samples was not processed in the same way as those in [8], which prevents us from performing direct comparisons on viral loads between studies. Of the fifteen tissue samples from Atlantic Coast *A*. *forbesi* analyzed in Hewson et al., the concentration of VP1 was relatively higher than those reported for *Mediaster aequalis*, *Pisaster giganteus*, *Pisaster brevispinus*, and *Patiria miniata* [8]. Many of the stars tested in our study showed similar or higher viral loads than the positive control obtained from the Hewson laboratory. It is likely, however, that a loss in copy number of VP1 may have occurred in the positive control DNA sample provided by the Hewson lab. On the other hand, we did not process the samples to enrich for viral DNA (as done in [8]), which may result in a dilution of target DNA values in our samples. Future research could focus on comparative analyses of tissue samples from different echinoderm species on the Atlantic and Pacific Coasts of the USA.

Based on anecdotal reports and our direct observations in Rhode Island, SSWD has had a significant impact on sea star populations in the Northeast USA. It should, however, be noted that some of the sea star population may have developed a resistance to SSWD. Recent reports (Fall 2015) have stated that some populations of *A*. *forbesi* around Rhode Island seem to be increasing. Similar reports from the Pacific Coast show an increase in recruitment of juveniles to certain areas where adults have been all but wiped out. It is unclear whether these stars are the result of spawning post-outbreak, or if some managed to survive the outbreak altogether [10]. In either situation, it will take years for these individuals to reach sexual maturity, and even longer until we can accurately assess the impacts on community structure. Well-coordinated monitoring efforts that characterize the prevalence of the disease through time in locations throughout the Atlantic Coast of the USA are needed to assess impacts on coastal ecosystems. These efforts would also aid in identifying populations not affected by the disease, providing material for better controlled challenge experiments that would contribute to characterization of the pathogenesis and etiology of the disease.

## Supporting information

S1 Table Citizen survey reports for presence/absence of SSWD. (XLSX)

S2 Table Data on sea stars tested for the presence of the SSaDV VP1. (XLSX)

S1 Text Sequences of DNA fragments from amplification of DNA samples from *Asterias forbesi* affected with SSWD using primers to the V4 region of SSaDV. (DOC)

## References

[1] Hyman LH. The invertebrates. Vol. 4 New York; London(etc): McGraw-Hill; 1955.

[2] Menge BA, Berlow EL, Blanchette CA, Navarrete SA, Yamada SB. The Keystone Species Concept: Variation in Interaction Strength in a Rocky Intertidal Habitat. Ecol Monogr. 1994;64: 249–286. doi: 10.2307/2937163

[3] MacKenzie J, Pikanowski R. A Decline in starfish, *Asterias forbesi*, abundance and a concurrent increase in Northern Quahog, *Mercenaria mercenaria*, abundance and landings in the Northeastern United States. Mar Fish Rev. 1999;61: 66–71.

[4] Lessios HA. Mass mortality of *Diadema antillarum* in the Caribbean: What have we learned? Annu Rev Ecol Syst. 1988;19: 371–393. doi: 10.1146/annurev.es.19.110188.002103

[5] Dungan ML, Miller TE, Thomson DA. Catastrophic decline of a top carnivore in the Gulf of California rocky intertidal zone. Science. 1982;216: 989–991. doi: 10.1126/science.216.4549.989 17809070 10.1126/science.216.4549.989

[6] Bates AE, Hilton BJ, Harley CDG. Effects of temperature, season and locality on wasting disease in the keystone predatory sea star *Pisaster ochraceus*. Dis Aquat Organ. 2009;86: 245–251. doi: 10.3354/dao02125 20066959 10.3354/dao02125

[7] Eckert GL, Engle JM, Kushner DJ. Sea star disease and population declines at the Channel Islands. Proceedings of the fifth California Islands symposium. 2000. pp. 390–393.

[8] Hewson I, Button JB, Gudenkauf BM, Miner B, Newton AL, Gaydos JK, et al Densovirus associated with sea-star wasting disease and mass mortality. Proc Natl Acad Sci. 2014;111: 17278–17283. doi: 10.1073/pnas.1416625111 25404293 10.1073/pnas.1416625111 PMC4260605

[9] Eisenlord ME, Groner ML, Yoshioka RM, Elliott J, Maynard J, Fradkin S, et al Ochre star mortality during the 2014 wasting disease epizootic: role of population size structure and temperature. Phil Trans R Soc B. 2016;371: 20150212 doi: 10.1098/rstb.2015.0212 26880844 10.1098/rstb.2015.0212 PMC4760142

[10] Montecino-Latorre D, Eisenlord ME, Turner M, Yoshioka R, Harvell CD, Pattengill-Semmens CV, et al Devastating transboundary impacts of Sea Star Wasting Disease on subtidal asteroids. PLOS ONE. 2016;11: e0163190 doi: 10.1371/journal.pone.0163190 27783620 10.1371/journal.pone.0163190 PMC5082671

[11] Menge BA, Cerny-Chipman EB, Johnson A, Sullivan J, Gravem S, Chan F. Sea Star Wasting Disease in the keystone predator *Pisaster ochraceus* in Oregon: Insights into differential population impacts, recovery, predation rate, and temperature effects from long-term research. PLOS ONE. 2016;11: e0153994 doi: 10.1371/journal.pone.0153994 27144391 10.1371/journal.pone.0153994 PMC4856327

[12] Fahsbender E, Hewson I, Rosario K, Tuttle AD, Varsani A, Breitbart M. Discovery of a novel circular DNA virus in the Forbes sea star, *Asterias forbesi*. Arch Virol. 2015;160: 2349–2351. doi: 10.1007/s00705-015-2503-2 26112764 10.1007/s00705-015-2503-2

[13] Evans AS. Causation and disease: The Henle-Koch postulates revisited. Yale J Biol Med. 1976;49: 175–195. 782050 PMC2595276

[14] Page K. Bone Bancroft JD and Stevens A “Theory and Practice of Histological Techniques.” New York: Churchill Livingstone; 1996.

[15] Gauger E, Gomez-Chiarri M. 16S ribosomal DNA sequencing confirms the synonymy of *Vibrio harveyi* and *V*. carchariae. Fish Anim Vet Sci Fac Publ. 2002; doi: 10.3354/dao052039 12517004 10.3354/dao052039

[16] Quinn RA, Metzler A, Smolowitz RM, Tlusty M, Chistoserdov AY. Exposures of *Homarus americanus* shell to three bacteria isolated from naturally occurring Epizootic Shell Disease lesions. J Shellfish Res. 2012;31: 485–493. doi: 10.2983/035.031.0208

[17] Omran N, Eissa S. Screening of microbial contamination and antimicrobial activity of sea cucumber *Holothuria polii*. Egypt J Aquat Biol Fish. 2006;10: 21–31. 10.1177/0748233712448116 22653870

[18] Ottesen EA, Marin R, Preston CM, Young CR, Ryan JP, Scholin CA, et al Metatranscriptomic analysis of autonomously collected and preserved marine bacterioplankton. ISME J. 2011;5: 1881–1895. doi: 10.1038/ismej.2011.70 21716310 10.1038/ismej.2011.70 PMC3223310

[19] Kohl WT, McClure TI, Miner BG. Decreased temperature facilitates short-term Sea Star Wasting Disease survival in the keystone intertidal sea star *Pisaster ochraceus*. PLOS ONE. 2016;11: e0153670 doi: 10.1371/journal.pone.0153670 27128673 10.1371/journal.pone.0153670 PMC4851418

[20] Burge CA, Eakin CM, Friedman CS, Froelich B, Hershberger PK, Hofmann EE, et al Climate change influences on marine infectious diseases: Implications for management and society. Annu Rev Mar Sci. 2014;6: 249–277. doi: 10.1146/annurev-marine-010213-135029 23808894 10.1146/annurev-marine-010213-135029

[21] Harvell CD, Kim K, Burkholder JM, Colwell RR, Epstein PR, Grimes DJ, et al Emerging marine diseases-Climate links and anthropogenic factors. Science. 1999;285: 1505–1510. doi: 10.1126/science.285.5433.1505 10498537 10.1126/science.285.5433.1505

[22] Thurber RV, Payet JP, Thurber AR, Correa AMS. Virus-host interactions and their roles in coral reef health and disease. Nat Rev Microbiol. 2017;15: 205–216. doi: 10.1038/nrmicro.2016.176 28090075 10.1038/nrmicro.2016.176

[23] Becker PT, Egea E, Eeckhaut I. Characterization of the bacterial communities associated with the bald sea urchin disease of the echinoid *Paracentrotus lividus*. J Invertebr Pathol. 2008;98: 136–147. doi: 10.1016/j.jip.2007.12.002 18191940 10.1016/j.jip.2007.12.002

